# Asthma prevalence, associated factors and role of adverse childhood experiences among school-going adolescents: the national School Health Survey in Togo (SHeST-study)

**DOI:** 10.1136/bmjresp-2025-003776

**Published:** 2026-03-03

**Authors:** Arnold Junior Sadio, Doevi Mawuena Mawuena Biaou, Pwemdeou Efalou, Fifonsi Adjidossi Gbeasor-Komlanvi, Innocent Gabkiangbe, Martin Kouame Tchankoni, Gérard Koglo, Gilbert Aku Agbetoglo, Boyodi Mewezinoh, Maïssala Zoutene, Liliane Derpeng Sam-Sam, N’dalna Berasngar, Yosua Aki, Balla Bilivogui, Atsu Koffi Aziagbe, Abdou Gafarou Gbadamassi, Tete Amento Stephane Adambounou, Didier Koumavi Ekouevi, Komi Seraphin Adjoh

**Affiliations:** 1 National Institute for Health and Medical Research (INSERM), Research Institute for Sustainable Development (IRD), Bordeaux Population Health Centre, UMR 1219, Université de Bordeaux, Bordeaux, Nouvelle-Aquitaine, France; 2 Faculty of Health Sciences, Department of Public Health, Center for Training and Research in Public Health, University of Lomé, Lomé, Togo; 3 African Center for Research in Epidemiology and Public Health (CARESP), Lomé, Togo; 4 Pneumology Department, Kara University Hospital, Kara, Togo; 5 Pneumology Department, Sylvanus Olympio University Hospital, Lomé, Togo

**Keywords:** Asthma Epidemiology, Paediatric asthma

## Abstract

**Background:**

Limited contemporary epidemiological data exist for asthma among school-aged adolescents in Togo. This study aimed to estimate the prevalence of asthma among Togolese adolescents in Grand-Lomé (an urbanised region in the south) and Kara (a more rural area in the north). Furthermore, we explored demographics and risk factors associated with asthma diagnoses, with specific emphasis on the role of adverse childhood experiences (ACEs).

**Methods:**

A cross-sectional study was conducted in secondary schools across southern and northern regions of Togo between February and March 2025. Adolescents aged 10–19 years were enrolled using multistage stratified random sampling. Asthma screening utilised International Study of Asthma and Allergies in Childhood (ISAAC) questionnaires and spirometry with reversibility testing. ACEs were assessed using ACE-Q. A multilevel mixed-effects penalised binary logistic regression model identified factors associated with asthma.

**Results:**

Among 2416 adolescents included in final analysis, median age was 16.0 years (IQR: 14.0–17.0), with 55.4% female. Overall asthma prevalence was 8.7% (95% CI: 7.6 to 9.9), with higher rates in the southern region (10.5%) compared with northern region (5.9%) (p<0.001). High childhood adversity (adjusted OR (aOR)=1.79; 95% CI: 1.09 to 2.94) was associated with asthma. Other factors significantly associated with asthma included: parental history of asthma (aOR=2.87; 95% CI: 2.03 to 4.05), and overweight/obesity (aOR=1.81; 95% CI: 1.22 to 2.68). Older age (aOR=0.93; 95% CI: 0.87 to 0.99) and male gender (aOR=0.67; 95% CI: 0.48 to 0.93) were associated with lower asthma likelihood.

**Conclusion:**

Asthma prevalence among school-going adolescents in Togo is substantial and shows marked regional variation. Beyond established risk factors, the observed association with ACEs supports the need for integrated strategies addressing both physical and psychosocial determinants and for strengthening school-based surveillance and care pathways.

**WHAT IS ALREADY KNOWN ON THIS TOPIC**

- Asthma prevalence in Africa has been rising, but data for Togolese adolescents were last recorded in the 1990s at 3%.

**WHAT THIS STUDY ADDS**

- This study reveals a current prevalence of 8.7% among this demographic, nearly tripling the earlier figure. It confirms associations between asthma, family history and overweight/obesity and identifies adverse childhood experiences as a relevant factor.

**HOW THIS STUDY MIGHT AFFECT RESEARCH, PRACTICE OR POLICY**

- These findings advocate for a national surveillance programme for respiratory diseases in Togo. They also highlight the need for public health policies and school interventions addressing modifiable risk factors like overweight/obesity and childhood trauma.

## Background

 Chronic respiratory diseases, including asthma, represent a growing global health burden. They disproportionately affect low- and middle-income countries, where their prevalence is closely linked to poverty, infectious diseases and other non-communicable diseases.^1^ Asthma is an incurable, yet manageable, respiratory disease characterised by chronic inflammation of the airways, leading to reversible partial bronchial obstruction.^2^ According to the latest Global Asthma Network estimates from 2019, asthma affected over 260 million people worldwide, with especially high prevalence and severity in resource-limited settings.^3^ Climate change, rapid urbanisation and increasing air pollution have contributed to rising exposure to environmental risk factors, further driving up asthma prevalence, particularly in urban areas of developing countries.^4^ Several studies have reported increased incidence of asthma and chronic bronchitis, notably in sub-Saharan Africa.^4^ Despite these trends, the epidemiology of asthma in Africa remains insufficiently documented, with available data often fragmented, especially among school-aged children and adolescents.^4 5^ A review, published in 2013, reported a steady increase in asthma prevalence among African children under 15 years, from 12.1% in 1990 to 13.9% in 2010.^6^ In South Africa, a study conducted between 2019 and 2021 among 3 957 adolescents reported a prevalence rate of 13.7%, higher than in the general population.^7^ A global study published in 2021 estimated that, on average, 15.4% of African adolescents exhibited asthma symptoms, compared with 10.4% worldwide.^8^ A systematic review and meta-analysis published in 2025 estimated the global prevalence of childhood asthma at 10.2%, with an African prevalence of 11%, reaching as high as 16% in Angola and as low as 6% in Nigeria.^9^ More recent regional studies highlight a significant gap in diagnosis. For instance, a survey in Nigeria and the Democratic Republic of Congo found that while 4.8% of adolescents had asthma symptoms, only 20.2% of them had ever received a formal doctor’s diagnosis.^10^ Echoing this, a large multicountry study conducted across six urban sites in sub-Saharan Africa, Malawi, South Africa, Zimbabwe, Uganda, Ghana and Nigeria, found significant variation in the prevalence of asthma symptoms, ranging from 4.2% in Malawi to 23.8% in South Africa.^11^ Crucially, this study revealed that among adolescents reporting severe asthma symptoms, a staggering 77.9% had never been clinically diagnosed.^11^ These findings underscore a high, often unmanaged, burden of severe asthma among adolescents in the region, who are consequently not receiving appropriate therapy.^10 11^

In Togo, available epidemiological data remain limited. A hospital-based study carried out in Lomé (the capital, located in the south) reported a hospital prevalence of 40.9% among children aged 6–15 years attending a pneumo-allergology unit between 2000 and 2007.^12^ A school-based survey conducted in 2018 in the Savanes region (in the north of the country) estimated the prevalence of asthma at 5.4% among children aged 6–15 years, with a predominance in boys.^13^ In Lomé, the last school-based survey among 1 861 children dates back to 1994 and reported a prevalence of 3.7%.^14^

While it is well established that asthma often begins in early childhood under the influence of genetic, immunological and early environmental factors,^5^ several less recognised factors may influence its onset and severity. The relationship between asthma and psychological distress is often bidirectional: while psychological distress can trigger or worsen asthma symptoms, asthma can conversely contribute to heightened anxiety and depression.^15 16^ A critical factor in this interplay is exposure to adverse childhood experiences (ACEs), which have been linked to an increased risk of developing asthma and the exacerbation of existing symptoms.^16 17^ This vulnerability is particularly pronounced in adolescents living in low-income settings, where exposure rates to ACEs are generally higher. For example, data from the World Mental Health (WMH) surveys reveal that African countries in the initiative reported higher prevalence rates of ACEs compared with other nations within the WMH framework.^18^ Given the higher prevalence of ACEs in these low-income settings, and knowing that the resulting chronic stress is strongly associated with asthma risk and can amplify other triggers,^16 17^ it is critical for asthma studies to document and investigate this powerful psychosocial factor. This approach is especially vital in contexts like sub-Saharan Africa, where sociocultural taboos and infrastructural gaps make acknowledging and addressing the impact of ACEs challenging.^19 20^ Beyond these psychosocial factors, overweight and obesity are also noted as major risk factors. They are associated with increased asthma risk, greater severity and poorer asthma control, with the connection involving mechanical, inflammatory and metabolic pathways.^21^

These established and suggested risk factors are potentially magnified by broader, ongoing environmental changes. Therefore, considering ongoing climate change, increasing urbanisation and the growth of the automobile fleet since the 1990s, it is likely that the prevalence of asthma has continued to rise in Africa, particularly in Togo. However, to date, no contemporary, nationwide data, particularly in the school setting, have been published in Togo. This study aimed to estimate the prevalence of asthma in Togolese adolescents in Grand-Lomé (urbanised region in the south) and Kara (more rural area in the north). Further, we explored demographics and risk factors associated with asthma diagnoses with specific emphasis on the role of ACEs.

## Methods

### Study design and setting

The School Health Survey in Togo (SHeST) was a cross-sectional study conducted in secondary schools across both the southern and northern regions of Togo between February and March 2025. Key study locations included Grand-Lomé, housing Lomé, the capital city, and Kara, situated approximately 450 km north of Lomé. Grand-Lomé is the most urbanised of the two regions. It has a large fleet of vehicles that mainly run on fossil fuels, as well as numerous industries.^22^ The region also has a higher population density than Kara.^23^ According to a 2022 study, Lomé’s population was at high risk of exposure to air pollution.^24^ This exposure was four to five times higher than the standard set by the WHO.^24^

### Study population and sampling

The target population comprised school-going adolescents in Togo. Eligibility criteria included the following: (1) age between 10 and 19 years; (2) regular enrolment in one of the selected schools; and (3) provide consent to participate in the study.

#### Sample size

The required sample size was estimated using a formula for a single proportion. Calculations were based on expected asthma prevalence derived from previous studies in Togo and sub-Saharan Africa, with an assumed prevalence of 10.0% for the southern region and 5.0% for the northern region.^9 12 14^ With a 95% confidence level, a 2% margin of error and an allowance for 10% of missing or unusable data, a minimum of 2500 adolescents was required (1 500 from the southern region and 1000 from the northern region).

#### Sampling strategy

The sampling frame was established using a comprehensive list of schools (mixed-gender education) in Togo provided by the Ministry of Primary and Secondary Education.

A multistage, stratified random sampling approach was employed to ensure proportional representation across education divisional areas in the study regions (Kara in the north and Grand-Lomé in the south). The study regions exhibited the following school distribution: (1) Kara: this region has a total of 275 schools. This comprises 223 public institutions (58 urban and 165 rural) and 52 private institutions (41 urban and 11 rural); (2) Grand-Lomé: this region has a total of 706 schools. This includes 117 public institutions (105 urban and 12 semirural) and 589 private institutions (535 urban and 54 semi-rural).

Stratification was based on two key criteria: school type (public/private) and geographic location (urban, semiurban and rural). Recognising that schools in Lomé have generally larger student populations than those in Kara, a total of 15 schools were selected for the study: 6 from Grand-Lomé and 9 from Kara. A complete list of these schools and their total number of students is available in online supplemental file 1.

The sampling proceeded in three stages: (1) stage 1 (schools): schools were randomly drawn from the Ministry’s complete list; (2) stage 2 (classrooms): classrooms were randomly selected within the chosen schools and (3) stage 3 (adolescents): adolescents were randomly selected from the chosen classrooms.

Finally, the total number of adolescents sampled from each divisional area and school was determined using a weighted calculation to ensure the proportional representation of each subgroup within the overall study population.

### Data collection

Two standardised questionnaires were developed and implemented using KoboToolbox software. The primary questionnaire focused exclusively on asthma, incorporating items from the International Study of Asthma and Allergies in Childhood (ISAAC) and spirometry results.^25^ The second questionnaire addressed other non-communicable diseases and included sections on (1) sociodemographic characteristics and medical history, (2) substance use and environmental exposures, (3) anthropometric measurements (weight/height) and (4) mental health and ACEs. The asthma-specific questionnaire was administered by previously trained pulmonology interns.

Spirometry tests were performed using a Spirolab-New (Medical International Research, Rome) portable ultrasonic spirometer by a pneumology resident physician. Procedures followed the American Thoracic Society and European Respiratory Society technical statement.^26^ After the explanation, the operator demonstrated the appropriate technique, and a first series of forced manoeuvres was performed to obtain at least three acceptable or usable forced expiratory volume in 1 s (FEV1) and forced vital capacity (FVC) measurements. Patients were in a sitting position, and a nose clip or manual nostril occlusion was used during all manoeuvres. Subsequently, bronchodilator responsiveness testing was performed by administering 400 µg of Salbutamol via a spacer, followed by three acceptable FEV1 and FVC measurements 15 min later.

### Operational definitions

#### Asthma

Asthma was defined based on a combination of clinical criteria and spirometry abnormalities. Adolescents were considered asthmatic if they met at least one of the following conditions: (1) known medical history of asthma; or (2) history of wheezing (whether lifetime, in the past 12 months, or after physical activity); associated with: (3) obstructive pattern or positive bronchodilator responsiveness test at spirometry.

Spirometry predicted values were based on Global Lung Initiative 2012 equations with ‘Black American’ ethnic parameter.^27^ Spirometry was classified as ‘obstructive pattern’ when FEV1/FVC ratio was <0.80 in individuals aged 16 years or younger,^28^ and <0.75 in older adolescents.^29^ The bronchodilator responsiveness testing was positive if an increase from baseline in FEV1 of ≥12% predicted was observed.^30^

#### Mental health disorders

Depression was assessed using the Patient Health Questionnaire modified for Adolescents (PHQ-9A),^31^ and anxiety was measured using the Generalised Anxiety Disorder 2-item (GAD-2) scale.^32^ A composite binary variable ‘mental health disorder’ was created. Adolescents were classified as positive (1) if they reported moderate to severe depression (PHQ-9A≥10) or moderate to severe anxiety (GAD-2≥3) and negative (0) otherwise.

#### Adverse childhood experiences

ACEs were documented using the Adverse Childhood Experiences Questionnaire (ACE-Q).^33 34^ Scores were categorised as follows: 0 ACEs (no reported adversity), 1–3 ACEs (low to moderate adversity), and ≥4 ACEs (high adversity).

#### Physical activity

Physical activity was assessed via a questionnaire adapted from the Youth Risk Behaviour Surveillance System (YRBSS).^35^ The measure reflected time spent engaging in physical activity during physical education classes, assigned a score from 0 to 7, with each increment representing an additional 15 min of activity. Higher scores indicated greater participation in sports during physical education sessions.

### Data analysis

Quantitative variables were presented as means and medians with their SD or IQRs, as appropriate. Categorical variables were described using frequencies and proportions. The proportion of adolescents with asthma was estimated with 95% CIs. Comparisons of proportions were made using the χ^2^ or Fisher’s exact test, while Student’s t-tests or Wilcoxon tests were used for quantitative variables.

A multilevel mixed-effects penalised binary logistic regression model was applied to identify factors associated with asthma, modelled as a binary outcome variable (1: present, 0: absent). Explanatory variables were selected based on scientific rationale and existing literature. Statistical significance was considered at p<0.05. All analyses were performed using R software version 4.3.2 (R Foundation for Statistical Computing, Vienna, Austria).

## Results

**Figure 1:**
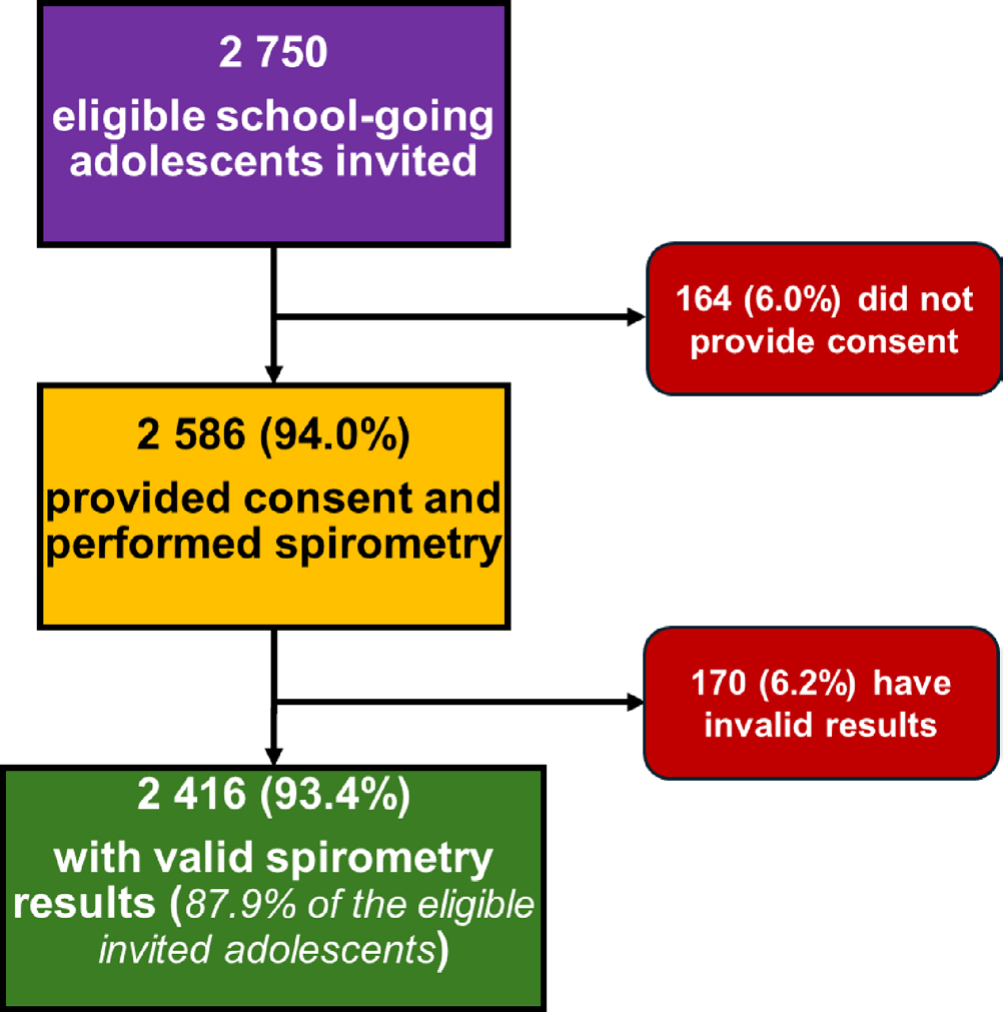
Flow chart of school-going adolescents’ inclusions, SHeST-study, Togo, 2025. SHeST, School Health Survey in Togo.

A total of 2750 eligible school-going adolescents were invited to participate in the study. Among them, 2586 (94%) provided consent and completed spirometry. Of those who underwent spirometry, 2416 (93.4%) had valid results and were included in the final analysis (figure 1). The median age of adolescents was 16.0 years (IQR: 14.0–17.0), and 55.2% were female.

### Asthma prevalence

**Figure 2:**
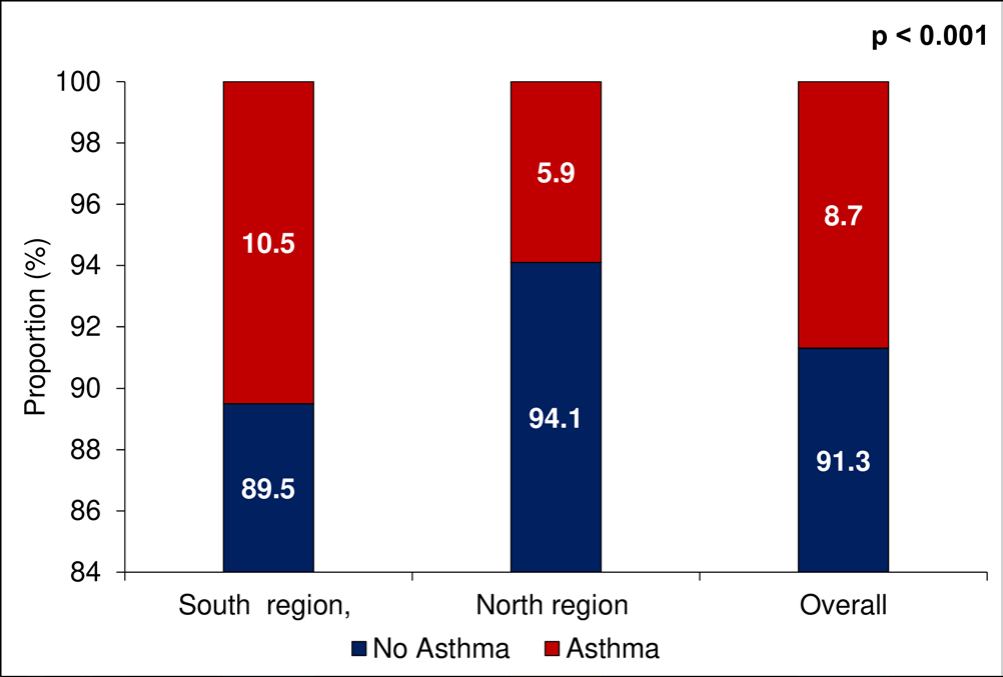
Asthma prevalence by region.

The prevalence of asthma was 8.7% (n=209/2416), 95% CI: 7.6 to 9.9. The southern region had a higher prevalence of asthma (10.5%, n=153) compared with the northern region (5.9%, n=56) (p<0.001) (figure 2).

### Sociodemographic, environmental and individual characteristics according to asthma status

Table 1 shows sociodemographic, environmental and individual characteristics of school-going adolescents, according to asthmatic status. The proportion of adolescents enrolled in private schools was higher among asthmatics (22.5%, n=47) than among non-asthmatics (12.6%, n=277; p<0.001).

**Table 1:** Sociodemographic, environmental and individual characteristics by asthma status among school-going adolescents in Togo, 2025^23^

	Asthma			
	Yes, n=209	No, n=2207	Overall, n=2416	P value
Age (years)				**0.025**
Median (IQR)	16 (14–17)	16 (14–17)	16 (14–17)	
Mean (SD)	15.26 (2.27)	15.62 (2.34)	15.59 (2.33)	
Gender, n (%)				**<0.001**
Female	144 (68.9)	1190 (53.9)	1334 (55.2)	
Male	65 (31.1)	1017 (46.1)	1082 (44.8)	
Region				**<0.001**
South	153 (73.2)	1306 (59.2)	1459 (60.4)	
North	56 (26.8)	901 (40.8)	957 (39.6)	
Type of school, n (%)				**<0.001**
Private	47 (22.5)	277 (12.6)	324 (13.4)	
Public	162 (77.5)	1930 (87.4)	2092 (86.6)	
History of parental asthma, n (%)				**<0.001**
No	150 (71.8)	1946 (88.2)	2096 (86.8)	
Yes	59 (28.2)	261 (11.8)	320 (13.2)	
Tobacco exposure, n (%)				0.855
No	154 (73.7)	1639 (74.3)	1793 (74.2)	
Yes	55 (26.3)	568 (25.7)	623 (25.8)	
Use of coal or firewood, n (%)*				**0.015**
No	21 (10.2)	129 (5.9)	150 (6.3)	
Yes	185 (89.8)	2062 (94.1)	2247 (93.7)	
(Missing)	3	16	19	
Furry pets at home, n (%)				0.104
No	106 (50.7)	983 (44.9)	1089 (45.4)	
Yes	103 (49.3)	1208 (55.1)	1311 (54.6)	
(Missing)	0	16	16	
Adverse childhood experiences, n (%)				**0.004**
No reported adversity	48 (23.0)	688 (31.2)	736 (30.4)	
Low to moderate adversity	119 (56.9)	1231 (55.8)	1350 (55.9)	
High adversity	42 (20.1)	288 (13.0)	330 (13.7)	
Mental Health disorders, n (%)				**0.002**
No	142 (67.9)	1712 (77.6)	1854 (76.7)	
Yes	67 (32.1)	495 (22.4)	562 (23.3)	
Weight status, n (%)				**<0.001**
Underweight	21 (10.0)	352 (15.9)	373 (15.4)	
Normal	142 (67.9)	1633 (74.0)	1775 (73.5)	
Overweight/obesity	46 (22.1)	222 (10.1)	268 (11.1)	
Physical activity				**0.011**
Median score (IQR)	2 (1–4)	3 (1–4)	3 (1–4)	
Mean score (SD)	2.69 (2.06)	3.01 (2.07)	2.98 (2.07)	

Bold p-values indicate a statistically significant difference (<0.05).

* Percentages are calculated based on the number of available responses for each variable.

A parental history of asthma was more common in the asthma group (28.2%, n=59) than in the non-asthma group (11.8%, n=261; p<0.001). There was no significant difference in exposure to tobacco smoke (26.3%, n=55 vs 25.7%, n=568; p=0.855). Household use of coal or firewood was widespread but slightly less frequent among asthmatics (89.8%, n=185) than non-asthmatics (94.1%, n=2062; p=0.015). The presence of furry pets at home did not differ significantly between groups (49.3%, n=103 vs 55.1%, n=1208; p=0.104).

Regarding ACEs, asthmatics showed a less favourable profile: high adversity was more frequent (20.1%, n=42 vs 13.0%, n=288) among asthmatics (p=0.004). Self-reported mental health disorders were also more prevalent in the asthma group (32.1%, n=67) than in the non-asthma group (22.4%, n=495; p=0.002).

### Clinical symptoms and spirometric abnormalities by asthma status

Table 2 summarises the distribution of clinical symptoms, atopic manifestations and spirometric results according to asthma status among school‐going adolescents.

**Table 2:** Clinical symptoms, atopic manifestations and spirometric abnormalities by asthma status among school‐going adolescents in Togo, 2025^23^

	Asthma (n=2416)			
	Yes, n=209	No, n=2 207	Overall, n=2416	P value
Lifetime chest wheezing, n (%)				**<0.001**
No	15 (7.2)	1837 (83.2)	1852 (76.7)	
Yes	194 (92.8)	370 (16.8)	564 (23.3)	
History of asthma, n (%)				**<0.001**
No	136 (65.1)	2120 (96.1)	2256 (93.4)	
Yes	73 (34.9)	87 (3.9)	160 (6.6)	
Dry cough at night in the past 12 months**,** n (%)				**<0.001**
No	119 (56.9)	1836 (83.2)	1955 (80.9)	
Yes	90 (43.1)	371 (16.8)	461 (19.1)	
Breathing difficulties, n (%)				**<0.001**
No	104 (49.8)	1876 (85.0)	1980 (82.0)	
Yes	105 (50.2)	331 (15.0)	436 (18.0)	
Itchy skin, n (%)				**<0.001**
No	155 (74.2)	1891 (85.7)	2046 (84.7)	
Yes	54 (25.8)	316 (14.3)	370 (15.3)	
Sneezing, or a runny or blocked nose, n (%)				**<0.001**
No	100 (47.8)	1618 (73.3)	1718 (71.1)	
Yes	109 (52.2)	589 (26.7)	698 (28.9)	
Itchy-watery eyes, n (%)				**<0.001**
No	100 (47.8)	1599 (72.5)	1699 (70.3)	
Yes	109 (52.2)	608 (27.5)	717 (29.7)	
Itchy throat, n (%)				**<0.001**
No	130 (62.2)	1851 (83.9)	1981 (82.0)	
Yes	79 (37.8)	356 (16.1)	435 (18.0)	
FEV1/FVC<normal, n (%)				**<0.001**
No	83 (39.7)	1876 (85.0)	1959 (81.1)	
Yes	126 (60.3)	331 (15.0)	457 (18.9)	
Positive bronchodilation test, n (%)				**<0.001**
No	93 (44.5)	1944 (88.1)	2037 (84.3)	
Yes	116 (55.5)	263 (11.9)	379 (15.7)	

Bold p-values indicate a statistically significant difference (<0.05).

FEV1, forced expiratory volume in 1 s; FVC, forced vital capacity.

The overall lifetime prevalence of chest wheezing was 23.3% (n=564). Among adolescents with asthma, 92.8% (n=194/209) reported ever‐wheezing versus 16.8% (n=370/2207) of those without asthma (p<0.001). Of the adolescents classified as asthmatic in this study, 34.9% reported having received a prior physician diagnosis of asthma, compared with 3.9% of the non-asthmatic group (overall 6.6%; p<0.001).

Spirometric abnormalities were markedly higher in the asthma group: 60.3% of asthmatics had an FEV₁/FVC ratio below the lower limit of normal versus 15.0% of non‐asthmatics (overall 18.9%; p<0.001), and 55.5% exhibited a positive bronchodilator response compared with 11.9% of their peers (overall 15.7%; p<0.001).

### Factors associated with asthma

Older age was significantly associated with a lower likelihood of asthma (aOR=0.93; 95% CI: 0.87 to 0.99) (table 3). Compared with females, males were less likely to have asthma (aOR=0.67; 95% CI: 0.48 to 0.93).

**Table 3:** Factors associated with asthma (mixed-effects penalised binary logistic regression models)

		Model 1		Model 2		Model 3	
		aOR (95% CI)	P value	aOR (95% CI)	P value	aOR (95% CI)	P value
Age (years)		0.95 (0.90 to 1.02)	0.138	0.93 (0.87 to 0.99)	**0.033**	0.93 (0.87 to 0.99)	**0.022**
Gender	Female	1		1		1	
	Male	0.55 (0.41 to 0.75)	**<0.001**	0.60 (0.43 to 0.82)	**0.001**	0.67 (0.48 to 0.93)	**0.016**
Region	South	1		1		1	
	North	0.69 (0.41 to 1.16)	0.162	0.72 (0.42 to 1.24)	0.234	0.73 (0.42 to 1.27)	0.269
Type of school	Private	1		1		1	
	Public	0.58 (0.34 to 1.00)	0.050	0.58 (0.34 to 1.00)	0.050	0.69 (0.40 to 1.21)	0.195
Parental history of asthma	No			1		1	
	Yes			2.95 (2.09 to 4.16)	**<0.001**	2.87 (2.03 to 4.05)	**<0.001**
Tobacco exposure	No			1		1	
	Yes			0.94 (0.66 to 1.34)	0.729	0.94 (0.66 to 1.33)	0.709
Use of coal or firewood	No			1		1	
	Yes			0.73 (0.43 to 1.23)	0.232	0.74 (0.44 to 1.25)	0.259
Furry pets at home	No			1		1	
	Yes			0.86 (0.63 to 1.17)	0.339	0.86 (0.63 to 1.17)	0.332
Adverse childhood experiences	No reported adversity			1		1	
	Low to moderate adversity			1.31 (0.92 to 1.88)	0.140	1.27 (0.88 to 1.83)	0.207
	High adversity			2.00 (1.26 to 3.18)	**0.004**	1.79 (1.09 to 2.94)	**0.022**
Mental health disorders	No					1	
	Yes					1.28 (0.90 to 1.82)	0.175
Weight status	Normal					1	
	Underweight					0.67 (0.41 to 1.10)	0.112
	Overweight/obesity					1.81 (1.22 to 2.68)	**0.003**

The education divisional areas and school were included in the model as clustering variables (random effects). Model 1 concerns sociodemographic factors, model 2 considers both sociodemographic and environmental factors and model 3 includes individual factors.

Bold p-values indicate a statistically significant difference (<0.05).

aOR, adjusted OR.

High childhood adversity (aOR=1.79; 95% CI: 1.09 to 2.94) was strongly associated with adolescent asthma. A parental history of asthma (aOR=2.87; 95% CI: 2.03 to 4.05) and overweight or obesity (aOR=1.81; 95% CI: 1.22 to 2.68) were also significantly associated with increased likelihood of asthma.

## Discussion

The present study aimed to estimate the prevalence of asthma among school-going adolescents in Togo and identify associated factors. Our findings revealed an overall asthma prevalence of 8.7% when combining clinical and spirometry criteria, with notable regional variations showing higher rates in the southern region (10.5%) compared with the northern region (5.9%). The study identified several significant associations between asthma and various factors, including ACEs, parental history of asthma, overweight/obesity, female gender and younger age.

### Global and regional prevalence comparison

The asthma prevalence observed in our study aligns with contemporary global estimates for childhood asthma. According to recent systematic reviews, the global prevalence of childhood asthma is estimated at 10.2% (95% CI: 9.5% to 11.0%), with an African prevalence of 11% (95% CI: 7% to 19%). Our findings place Togo within this continental range, though below the African average. Notably, prevalence varies considerably across African countries, ranging from 6% in Nigeria to 16% in Angola. These variations may be attributed to the methodological approaches employed for asthma screening or diagnosis. For instance, the South African study, which relied solely on ISAAC/Global Asthma Network methodology questionnaires, reported a prevalence of 13.7%, which was higher than our findings. Regional contextual factors can also explain these differences, as is the case in this study. Indeed, the regional disparities observed, with the southern (the most urbanised) region showing nearly double the prevalence of the northern region, reflect patterns commonly observed across Africa where urban areas tend to have higher asthma rates due to increased environmental pollutants, lifestyle changes and reduced exposure to protective rural environmental factors.^36 37^

In our study, the definition of asthma combined current symptoms with a self-reported personal history of asthma. This means that adolescents who had already been identified and labelled as having asthma by health professionals were more likely to be classified as asthmatic in our survey. From this perspective, the higher number of asthma cases observed in Grand-Lomé compared with Kara can partly be explained by differences in access to and use of health services, and in opportunities for prior diagnosis. According to the national health statistics report in Togo, Grand-Lomé concentrates a larger absolute supply of health personnel and health facilities than Kara, including more specialised structures and a denser urban health network.^38^ This configuration increases the probability that adolescents with recurrent respiratory symptoms encounter services where asthma can be recognised and communicated as such to families and adolescents. National statistics also show high outpatient service use in both regions, with a service utilisation rate of 69.6% in Grand-Lomé, suggesting frequent contact with care and multiple clinical encounters where respiratory conditions can be assessed.^38^ In this context, the combination of a denser health workforce and greater absolute service availability in Grand-Lomé could also have contributed to more adolescents having been previously diagnosed or informed that they have ‘asthma’. Given our definition, which includes personal history, this may partly explain the higher number of asthma cases recorded in Lomé during the survey.

### Temporal trends in Togo

Of particular significance is the comparison with historical data from Togo. The 1994 school-based survey in Lomé reported an asthma prevalence of 3.7% among school children.^14^ While acknowledging methodological differences between studies, our current findings suggest a potential threefold increase in asthma prevalence in the southern region over three decades. This temporal trend, if confirmed, would be consistent with global patterns showing rising asthma prevalence, particularly in developing countries experiencing rapid urbanisation.

This apparent increase underscores the urgent need for establishing a comprehensive surveillance programme for chronic respiratory diseases in Togo. The identification of potentially modifiable risk factors provides a foundation for targeted interventions that could help mitigate the rising burden of asthma among Togolese adolescents.

### Modifiable associated factors

#### Adverse childhood experiences and asthma

Our study identified a significant association between high childhood adversity and asthma. This finding is consistent with growing scientific literature demonstrating the relationship between ACEs and respiratory health outcomes. The mechanisms underlying this association are complex, involving chronic stress triggering inflammatory pathways and dysregulating the hypothalamic–pituitary–adrenal axis, leading to altered immune responses and increased susceptibility to respiratory conditions.^39 40^ In addition to these biochemical pathways, asthmatic children exposed to adverse family circumstances may experience suboptimal healthcare access and clinical monitoring. From a practical standpoint, ACEs should be regarded as potential cofactors operating in conjunction with genetic and environmental determinants through complex interactive mechanisms. These findings highlight the imperative for trauma-informed public health approaches to asthma prevention and management. Therapeutic strategies must extend beyond traditional medical interventions to include integrated social protection frameworks, family support systems and community-based programmes targeting ACE reduction. Healthcare providers and educational institutions require adequate resources to effectively identify and support adolescents experiencing adversity.

#### Overweight/obesity and asthma

The significant association between overweight/obesity and asthma observed in our study corroborates extensive international research. Excess weight is strongly associated with increased asthma risk, greater severity of the disease and challenges in achieving effective control.^41^,^43^ These effects are mediated through various mechanisms, including mechanical impacts on respiratory function, inflammatory pathways driven by adipokines and metabolic imbalances influencing airway responsiveness.^21 41^ The prevalence of overweight/obesity in our study population (11.1%) represents a concerning public health issue. Notably, this condition follows the same geographical pattern as asthma, with higher rates in the southern region (13.8%) compared with the northern region (7.0%). This parallel north-south gradient for both overweight/obesity and asthma prevalence reinforces our findings of a significant association between excess weight and asthma, suggesting that nutritional status may partially explain the geographical disparities in asthma prevalence. These findings emphasise the need for integrated approaches addressing both respiratory health and nutritional status through school-based nutrition programmes, community sports initiatives and coordinated care models that address both conditions simultaneously.

### Non-modifiable associated factors

#### Parental history of asthma

Our study showed a strong association between parental asthma history and adolescent asthma, consistent with decades of research demonstrating the substantial genetic component of asthma susceptibility.^44^,^46^ Twin studies and family-based genetic analyses have consistently shown that asthma has a heritability of approximately 60%–80%, making family history one of the strongest predictors of asthma development.^45^,^48^ From a public health policy perspective, the strong familial aggregation suggests the importance of family-based screening and prevention strategies. Healthcare systems should implement protocols for systematically assessing family history of asthma in paediatric care settings, with children having positive family histories considered for enhanced monitoring and early intervention programmes.

#### Age and gender associations

Our findings revealed that younger age and female gender were associated with increased asthma risk among adolescents. The female predominance reflects the well-documented gender shift that occurs during puberty, attributed to hormonal changes with oestrogen potentially influencing airway inflammation and responsiveness.^49 50^ These demographic patterns underscore the importance of implementing gender-sensitive programmes that address the unique needs of children and adolescents during their early developmental stages. Such initiatives can play a crucial role in shaping equitable asthma prevention and management strategies.

### Originality and limitations

This study represents one of the first comprehensive investigations of asthma prevalence and associated factors among school-going adolescents in Togo. The study stands out for its clinical assessments paired with spirometry and the use of validated international tools. It further explores underexamined links, such as ACEs and asthma, within the Togolese context.

Several limitations should be acknowledged. The absence of fractional exhaled nitric oxide (FeNO) measurements may have underestimated asthma prevalence, as FeNO is a valuable biomarker for airway inflammation. Social desirability bias likely led to underreporting of ACEs and tobacco exposure while overreporting physical activity levels. Despite these limitations, the study provides valuable baseline data for establishing a national surveillance programme for chronic respiratory diseases, such as asthma in school settings.

## Conclusion

Asthma prevalence among school-going adolescents in Togo is relatively high and shows marked regional variation, reflecting a complex interplay of modifiable and non-modifiable factors. The apparent threefold increase in prevalence since the 1990s highlights an urgent need for action. While our study confirms established risk factors like parental history and obesity, it identifies a significant association with ACEs in a sub-Saharan African context. This finding is particularly important as it links a taboo subject to a common physical illness, creating an opportunity to break down barriers and foster integrated health strategies that address both psychosocial trauma and respiratory health. Therefore, policies must both target modifiable factors like overweight/obesity and also prioritise trauma-informed, school-based and community-wide interventions. Establishing systematic surveillance is essential to monitor these trends and guide efforts to reduce the burden of asthma on Togolese adolescents.

## Supplementary material

10.1136/bmjresp-2025-003776 online supplemental file 1

## Data Availability

Data are available upon reasonable request.
