# Supplementary material for: Asthma prevalence, associated factors and role of adverse childhood experiences among school-going adolescents: the national School Health Survey in Togo (SHeST-study)
Source: BMJ Open Respir Res. 2026 Mar 3;13(1):e003776. doi: 10.1136/bmjresp-2025-003776 (PMC12958903; doi:10.1136/bmjresp-2025-003776)
Supplement: online supplemental file 1 [file bmjresp-13-1-s001.docx]

**Supplementary file 1: list of selected schools**

| **Region** | **EDA*** | **Location** | **Schools** | **Students number** | **Sample** |
| --- | --- | --- | --- | --- | --- |
| **North (Kara)** | | | | **8 539** | **1 000** |
|  | Bassar | Rural | Kidjaboun HS | 823 | 96 |
|  |  |  | Kabou-West GEC | 782 | 92 |
|  |  | Urban | Bassar HS | 1 167 | 137 |
|  | Kara | Rural | Ketao GEC | 1 140 | 134 |
|  |  |  | Lassa Elimde GEC | 785 | 92 |
|  |  | Urban | Kara Tomde HS | 1 455 | 170 |
|  |  |  | Chaminade college** | 604 | 71 |
|  | Niamtougou | Rural | Broukou HS | 858 | 100 |
|  |  | Urban | Kante HS | 925 | 108 |
| **South (Grand-Lomé)** | | | | **10 707** | **1 500** |
|  | Agoe | Urban | NDE Vakpossito College** | 1 008 | 141 |
|  |  |  | Agoe HS | 2 508 | 351 |
|  |  | Semi-Urban | Nanegbe HS | 1 451 | 203 |
|  | Golfe | Urban | Protestant college** | 1 119 | 157 |
|  |  |  | Adidogome I HS | 2 586 | 362 |
|  |  | Semi-Urban | Avedji-Elavagnon HS | 2 035 | 285 |
| **Total** |  |  |  | **19 246** | **2 500** |

*EDA: *education divisional areas*; GEC: General Education College; HS: High school; *Private (based on the global number of students)
